# In-verse reflection: structured creative writing exercises to promote reflective learning in medical students

**DOI:** 10.1007/s10912-022-09740-7

**Published:** 2022-05-20

**Authors:** David McLean, Neville Chiavaroli, Charlotte Denniston, Martin Richardson

**Affiliations:** 1 Epworth Clinical School, Melbourne, Australia; 2 Australian Council for Educational Research, Melbourne, Australia; 3 Department of Medical Education, Melbourne Medical School, University of Melbourne, Melbourne, Australia

**Keywords:** Reflective writing, Indirection, Medical Humanities, Medical Students, Playful learning

## Abstract

Medical educators recognize the value of reflection for medical students and the role creative writing can play in fostering this. However, direct creative writing tasks can be challenging for many students, particularly those with limited experience in the arts and humanities. An alternative strategy is to utilize an indirect approach, engaging students with structured tasks that obliquely encourage reflection. This paper reports one such approach. We refer to this approach as *in-verse reflection*, playing on both the structure of the writing and its novel approach to reflection. Students were invited to write, in verse-like structures, about their personal and clinical experiences as medical students. Thematic analysis of their creative outputs and reactions identified four principal themes: the challenges of life as a medical student, the emotional demands of the medical course, a sense of connectedness and solidarity with fellow students, and a sense of marginality within the hospital system. Students generally found the tasks highly engaging and conducive to reflection, producing texts representing significant insights into their experiences as medical students. The reported method offers a relatively simple, structured, and guided approach to reflective writing, adding to the repertoire of methods available to educators in the medical humanities.

## Introduction

Reflective writing is increasingly seen as an important educational practice in medical and health professional education to help achieve desired learning outcomes such as communication, empathy, and professionalism (Moniz et al. 2015). Medical educators may use a variety of writing tasks and forms to engage students and foster reflection, including focused essays, journal entries, and creative writing tasks (Green et al. 2016; Kerr 2010). While the potential value of reflective writing is widely acknowledged, unstructured approaches may be hampered by student reluctance or hesitation to engage or participate meaningfully (Aronson 2011; Sandars 2009). In particular, the individual free writing approach typical of many written exercises may not align with contemporary students’ preference for group-based and creative activities (Sandars 2009). On the other hand, tightly structured or focused reflective writing tasks, especially those that are summatively assessed, may be viewed cynically by students, who often aim to give teachers the responses they think educators are looking for (Belling 2011; Birden and Usherwood 2013).

In our second-year medical program, we initiated an approach to guide and promote student reflection of their clinical learning experiences, the implementation of which was feasible for educators and genuinely engaging for students. To do so, we drew on short and structured creative writing exercises with the aim of guiding and prompting students to think about their professional journeys and experiences. Our approach is based primarily on the first author’s educational practice of using such activities in English classes in a secondary school setting (McLean 2020). This approach has been relatively under-utilized in the creative writing practices currently employed and represented in medical education (Bolton 1999; Cowen et al. 2016; Kerr 2010; Morris 2001). As many educators have pointed out, explicit creative writing tasks can form barriers for students for several reasons, including the belief that they do not have the necessary writing skills, an unease about exercises that have no single correct response, or a discomfort with emotionally laden issues (Kerr 2010; Sandars 2009; Shapiro et al. 2009). This arguably applies even more so to *poetic* writing, where the ambiguity and fluidity of language and meaning can significantly deter students unfamiliar with the genre (Wellbery 2006). However, as Johanna Shapiro has shown in her book *The Inner World of Medical Students* (2009), there are numerous medical students who are (or become) very comfortable and proficient in reflecting through poetry and verse and who derive great benefit and meaning from engaging in this format. For them—and potentially their peers—the poetic form can be liberating and enabling.

Our approach aims to draw on the power of poetic form and ideas while attempting to address the challenges it can present to medical students. We generate short, simple, and structured tasks which, while not presented as poetry as such, do produce writing with a poetry-like structure. We refer to this method as *in-verse reflection*, playing on both the structure of the writing and its indirect approach to reflection. In this method, the focus is (seemingly) on the structured sequence of instructions rather than the creative process/product; the reflective component occurs almost incidentally, although no less significantly, through both the activity itself and the ensuing collaborative discussion. Through such seemingly trivial writing tasks, we engage students who are usually outcome-oriented and assessment-driven in creative and fun activities that can, nevertheless, lead to insightful and often quite profound writing and reflection. Essentially, we aim to awaken reflection in students instead of attempting to guarantee it through more direct and potentially constrained approaches (Saeverot 2022). It is, in some ways, a form of gentle misdirection—or, speaking more pedagogically, it uses *indirection* as a way of fostering reflection.

The use of indirect techniques and pedagogies has a strong base in both education and humanities disciplines, where indirection is defended as frequently desirable, if not necessary, to generate reflective insights and meanings that may be limited by direct communication or transmission of knowledge (Fraser 2020; Saeverot 2022). The theory of indirection is notably, and more popularly, represented in the book *Drawing on the Right Side of the Brain* by Betty Edwards (1979), which teaches drawing through the strategy of turning the figure upside down and forcing the brain to forego the assumptions and expectations of conventional orientation, bringing intuitive and spontaneous elements to the fore. The use of indirection is also not new in medical education. Both Bleakley (2015, 146) and Belling (2011) report on the use of indirection in “art rounds” in medical contexts. As Belling explains, commenting on the research study of Gaufberg and Williams (2011), museum objects were used to promote reflection in medical students by focusing primarily on the art object *itself* rather than the transferable skills. While such skills-based approaches are not uncommon in the medical humanities (Blease 2016; Chiavaroli et al. 2018), they can come unstuck in the context of teaching for reflection. As Belling argues, “authentic personal responsiveness” is integral to reflection (2011, 580), and overly didactic approaches to teaching it, even when using creative products, can inadvertently elicit superficial or even cynical responses. Furthermore, drawing inspiration from Emily Dickinson’s notion of telling the truth *slant*, some medical educators seek to use poetry’s natural affinity for indirection to enable students to produce experiential insights about their professional development (Gaufberg and Batalden 2007; Shapiro and Stein 2005). Such an approach, Shapiro and Stein write, “allows learners to more easily examine intangible aspects of their relational experiences in medical school. Issues that seem straightforward when organized through the well-defined and prescribed formulas of the case presentation yield other interpretations when explored in verse” (2005, 279). In the study reported here, we sought to utilize these very advantages of verse writing and indirect reflection by using creative tasks that were more guided and structured than might typically be the case with poetry sessions.

## Methods

The second year of the medical course marks the transition from a pre-clinical campus-based first year to clinically based learning for the graduate-entry Doctor of Medicine (MD). The Epworth Hospital is one of the smaller clinical schools of the Melbourne University Medical School, with approximately 15–20 students based at the hospital for their second-year clinical rotations (from a full second-year cohort of approximately 350 students). As such, there was an opportunity to engage with students in creative reflective exercises that may not have been possible in larger cohorts. Our aim was to enable reflection on clinical learning experiences through creative exercises and to balance the otherwise dominant science basis of the clinical curriculum while introducing students to alternative ways of knowing in medicine, such as those associated with the medical humanities (Chiavaroli et al. 2018; Jones et al. 2019). The cohort of second-year MD students from the Melbourne University Medical School based at the Epworth Hospital was invited to participate in four one-hour workshops. The workshops reported in this paper were conducted throughout the course of 2019. Ethics approval for the study was obtained from the Human Research Ethics Committee, Melbourne Medical School, University of Melbourne.

The workshops consisted of several writing tasks designed to stimulate creative responses about students’ clinical education experiences. They were not cumulative or sequential in orientation, though each provided an opportunity for students to take a more holistic view of their experience. Participation was entirely voluntary, and there was no assessment attached to the workshops. Sessions were scheduled during March, May, August, and October to coincide with the timing of different clinical rotations (namely, Foundation, General Medicine, Surgery, and Emergency Medicine). Table 1 below outlines the nature of the tasks used in each workshop.

**Table 1.** Workshop focus and tasks

Workshop	Focus	Sample Task
1	Medical studies	Write down a noun that sums up your impression of your medical studies. Fold the top of the sheet over that word. Pass the sheet to your neighbor on the right. Now write down two adjectives that describe the noun you originally wrote. Fold the sheet again and cover those words. Pass the sheet along. Now write down three verbs that sum up the actions associated with medical studies. Fold and pass the sheet along again. Write a phrase that sums up medical studies for you. Fold and pass along again. Now write a synonym for the very first noun you wrote. Unfold the sheet and read what has been composed.
2	The latest rotation	Write a line in the middle of the page associated with your current clinical rotation. Now add another line, either preceding or following what you previously wrote. Now double the number of lines again by adding lines before, between, or after what you have already written. You can edit previous lines to help with the flow. Continue until there are eight lines, then try for sixteen if you still have time.
3	What you’ve heard	In a group, write down the phrases you’ve heard from doctors during the course of your current rotation. Now take these phrases and arrange them in any order you like. You can repeat lines, edit lines, and add other comments you’ve heard doctors use. Now repeat the task, this time focusing on what you’ve heard patients say. Finally, repeat the task once more, this time writing down what you’ve heard each other say in the common room.
4	Looking back	Think of an event from this year. Without naming the event, associate it with a feeling or feelings. These can be feelings you’ve witnessed or felt yourselves. Now think of an image or comparison to accentuate that feeling (i.e., a simile or metaphor). Next, think of a smell, sound, taste, touch, and sight that can be linked with that experience. Aim for at least two of the senses.

At the completion of each session, students were invited to share with the group the writing they had produced. Not all students chose to do so, but the majority at each workshop did. Students were also asked to provide a few lines reflecting on the nature of the activity in which they had participated. These reflections were anonymously written and collected in such a way that would not identify students while still providing useful evaluative reflections about the activities for us as educators. Each workshop, therefore, produced both creative products and explicit reflections from each student on the activities by way of workshop outputs.

The authors analyzed the collected data for prevalence and significance, following the protocol for qualitative thematic analysis outlined by Braun and Clarke (2006). All authors read through the data independently, coding for significant ideas and collating relevant data into key themes, and then met to compare and discuss codes and resultant themes. All authors discussed and debated the allocation of codes and their merging into broader themes until agreement was reached on the main themes presented below (Table 3). Although analysis commenced at the end of the first task, results were not used to modify subsequent tasks, which had already been planned and developed.

## Results

Fifteen medical students from the 2019 cohort participated in at least one workshop; eight students attended two sessions, and four attended three sessions. No student attended all four sessions. In total, 51 discrete creative products were generated by the participating students. Sample creative pieces are shown in Table 2 below. These are presented here solely as examples of the kind of writing produced through each task rather than as representing any particular theme or quality.

**Table 2.** Sample writings produced by students

Task 1 (Medical studies; collaborative task)	Task 2 (The latest rotation)
Interaction Time consuming, annoying Fretting, crying, struggling Lifelong learning for optimal patient care Apprentice	I don’t think I know anything much. I wanted to pull an all-nighter Thankfully I went to bed early last night Something that I don’t always do I woke up had breakfast then I went to the ED On Wednesday morning I saw a patient with epigastric pain. She was a nice old lady We had so many stories to tell I sat next to her bedside And we spoke for 2 hours Just about anything under the sun I wish I could do something more for her.
Task 3 (What you’ve heard)	Task 4 (Looking back)
Wait, what year are you in? Have you done this before? So, what do you think? Don’t answer a question with another question. It’s just practice, it comes with time. Follow a structure. They don’t teach you anatomy anymore? Use a systematic approach. Things were much harder when I was a student. They’re raising doctors these days to be soft. Wait until you’re a resident.	I didn’t expect to feel like I belonged, like a key in its lock. It was as if it fell into place. The heat of the lights overhead and the smell of disinfectant should have put me off. The image of shiny white bone was new but the feeling of cold steel in my hand grounded me. Four rings of metal on metal and it was done; we moved on to the next step.

Alongside the creative pieces, the students also provided 32 anonymous comments about their experiences in participating in the workshops. This provided evaluative data about the impact of the workshop, albeit at the level of student reactions only (Kirkpatrick 1996). Through the thematic analysis of students’ written products and reflections on the activities, we identified four key themes about students’ clinical learning experiences:

- the challenges of life as a medical student;
- the emotional demands of the medical course;
- a sense of connectedness and solidarity (with fellow students); and
- a sense of marginality (within the hospital system).

In addition to the above course-related themes, students’ evaluative comments on the nature of the creative activity itself were collated into a separate theme of Student Reactions. These themes are illustrated in Table 3, with representative comments drawn from students’ evaluative comments. Again, these examples are intended as illustrative only.

**Table 3.** Emerging themes from student outputs and reflections

Theme	Representative student evaluative comments
Challenges of life as a medical student	“The pieces my colleagues wrote about the struggles and joys of medicine and daily life were so close to the issues I experience myself.” (C19) “This exercise has allowed me to explore the challenges I have faced and the difference between my expectations and the eventual reality of MD2s and the hospital environment.” (G19) “Reminded me of the sacrifices we’ve made to get here to struggle with the stress and pressures.” (L19)
Emotional demands of the medical course	“My thoughts focused on experiences/events which elicited strong emotions, both good and bad.” (J19) “This exercise today forced me to consider the visceral effect of the experiences I’ve had so far.” (I19) “I have kept myself busy to avoid detesting medicine over my dissatisfactions. I have an awful impression of doctors.” (M19)
Sense of connectedness/solidarity (with fellow students)	“I feel more connected to my peers in some small way now, which for me is the greatest benefit.” (E19) “It showed me that we didn’t really share some aspects with each other, even though we had the same experiences. I thought I was on my own for those!” (F19) “I find it very reassuring that we are all going through similar things and my experience is not unique.” (I19) “Made me think and be aware of what I have done in the day and appreciate life a little more and know how my friends are doing and see that we are in the same boat.” (N19)
Sense of marginality (within the hospital system)	“Medicine can be quite isolating as you’re never quite sure if anyone else is having the same struggles and doubts you are.” (I19) “Frustrating to be reminded of the small role we play in the healthcare system.” (L19) “I guess one of the reasons I have avoided exploring this previously is that it is really conflicting to become someone, a doctor, that I have always disliked. I am unsure whether I will ever come to terms [with it].” (M19)
Student reactions (to the exercise)	“The activity was fun.… As someone who does not put much thought into more artistic pursuits, I found this to be a very good way to put my thoughts into a different order than they would usually be in.” (C19) “I found it somewhat difficult to think on the spot, as potentially with more time to reflect I may have come up with slightly different responses.” (D19) “Reflective practice has been unexpectedly fun. It’s interesting to see what comes to mind spontaneously for different people.” (E19) “This exercise has given me the insight into my first term and my approach to the transition… It has forced me to put things into perspective, away from the world of lectures, illness and medicine and consider how I fit into this framework.” (G19) “The activity was interesting as by adding lines in stages it meant I didn’t think about what I was writing. I was more focused on making it make sense than the content so I think the thoughts were more sincere.” (J19) “I enjoyed the session however I’m unsure what was being gotten at. It was fun to participate but I’m unsure of the relevance of this to my studies in medicine.” (O19)

## Discussion

For many medical educators with backgrounds in the humanities or a deep appreciation of the arts, the idea of using creative activities to help students write and reflect seems quite intuitive. This works well for the many medical students who have experience in such curricula and activities; for other students, however, the road to medical school has been paved with scientific textbooks and long hours of rote learning. Creative writing or reflection may not feel or come naturally in such a context. As many educators have noted (Kerr 2010; Sandars 2009; Shapiro et al. 2009), many students do not see themselves as writers or struggle to know what to write about in conventional reflection exercises. And, of course, the verse form itself is an unfamiliar and potentially intimidating genre for many medical students. In presenting the verse structure in such a structured and somewhat mechanical way, we encouraged and enabled our students to write and think quite differently from the objective, convergent ways more commonly utilized in the medical curriculum, aided by the apparent freedom of the indirect approach to reflection.

Despite some initial hesitation and uncertainty, students engaged positively and collaboratively with the activities. Part of this engagement is undoubtedly attributable to the voluntary nature of participation in the workshop, but the challenge and unfamiliarity posed by these tasks should not be underestimated. Both the nature of the activities and openness of the tasks were very different from the type of logico-deductive ways of thinking and factual scientific content that dominate the medical curriculum (Bleakley 2015), and several students noted this in their evaluative comments (e.g., “I have not approached reflection in this format before. … reflection does not have to be incredibly time consuming or daunting” [I19]). We believe the constrained and structured set of instructions provided important focus and guidance to the students (Aronson 2011), enabling them to overcome initial uncertainties and produce verse-like compositions that appeared to meaningfully represent their own clinical experiences while also resonating deeply with their peers. Many of the students’ verses were insightful and highly evocative; we would even say *poetic*, though this was not the point of the exercise.

From the students’ evaluative comments, it was clear that the commonality of their experiences and reactions evoked a strong sense of solidarity and relief, as others were experiencing similar feelings about the course and their sense of emerging professional identities. Many students mentioned the sense of camaraderie among the students during the exercise, and several comments related to the *affective* dimension of the exercise. As one student remarked: “The thought-provoking nature of these sessions has allowed me an opportunity to re-appreciate the exquisiteness of abstraction. I forgot how interesting things are” (B19). Many students reported that the tasks were actually *fun*—certainly more fun than they had anticipated—and something not necessarily associated with a medical course. Even those students who struggled somewhat with the indirectness of the tasks (e.g., students D19 and O19 in Table 3) still generally responded positively to the sessions.

We see other connections between the in-verse reflection approach and the broader project of the humanities in medical education, besides the use of indirection. The humanities continue to be a source of renewal and diverse pedagogies for medical curricula, being utilized in various ways and for different purposes. Initially, its primary role was to support the learning of clinical skills (Blease 2016), such as communication, empathy, and teamwork. Medical humanities scholars have extended this scope to more epistemological rationales that include clinical reasoning and personal identity formation (Bleakley 2015; Boudreau and Fuks 2015; Chiavaroli 2017; Moreno-Leguizamon et al. 2015) as a counterbalance to the dominant scientific and technological foundation of medical practice (Montgomery 2006; Whitehead 2013). We see the in-verse reflection approach as applicable to both instrumental and epistemological orientations depending on the emphasis placed on the activities as either a means of facilitating reflection or a broader way of prompting students to think differently about their clinical learning experiences.

Another connection with pedagogical practice in the humanities is the notion of “playful learning,” a relatively well-utilized pedagogy in school contexts (Kangas et al. 2017; Mardell et al. 2019, 232) and one that is emerging in higher education (Forbes 2021), especially in the humanities disciplines (Jensen et al. 2022). We had certainly hoped that our students would find the activities fun, but we were surprised at the number of comments that reflected enjoyment even alongside confusion or bewilderment. Some students even appreciated the humor inherent in the approach (e.g., “Adding humor is therapeutic in reflection and allowed me to overcome thoughts and experiences that were previously avoided or swept under the rug” [F19]). Of course, ours is not the only method to draw on this element of humanities pedagogy; a similar underlying spirit can also be seen in the successful use of comics and other creative practices in medical education (Green 2013; Shapiro et al. 2021; Maatman et al. 2021). Such playful learning can be a valuable counter to the typical emphasis on the “logics of efficiency, competition and achievement” (Jensen et al. 2022, 206) that can characterize many medical courses, while other research suggests that play promotes learning and engagement and helps create relational safety and positive affect and motivation (Forbes 2021). Jensen et al. (2022) go further to draw more direct links with humanities pedagogies:

Addressing teaching activities as playful relates to broader aspects of humanities in higher education that aim to support the students’ development of judgment and active engagement in learning; of their individual, professional and social identity; and of meaningful life choices within and beyond their education. (199)

These are exactly the kind of broad epistemological perspectives and cognitive skills we want our medical students to acquire, alongside the necessary and obviously important scientific and clinical knowledge required for good medical practice. The in-verse reflection method appears to tap into this vein of playful learning, providing an adaptable and useful framework for incorporating such an approach into medical education. Even with the relatively limited sessions and non-compulsory participation, the mix of serious insight with lighthearted and occasionally wry sentiments is a marked feature of the students’ outputs.

Nonetheless, we recognize that many students may find the indirect and playful nature of the tasks potentially irrelevant or even disconcerting. While this can be a useful source of “creative tension” in reflective activities (Wald 2015, 702), it may also point to the challenges of trying to balance an entire science-focused curriculum with a few short voluntary creative sessions. As educators, we certainly need to acknowledge and respect that not all students will warm to such activities, but our results encourage us that most students are prepared to give it a genuine try. Ultimately, though, we see the in-verse approach as an additional method for engaging students in reflective practice to be used alongside more direct and conventional approaches to facilitate reflection in medical students.

Several other limitations of this study are also acknowledged. The number of participants was relatively small and limited to a single clinical learning site. The site itself may also limit generalizability in the sense that, as the most recently instituted clinical school of the MD program, there may well be an openness to innovative approaches that may not be easily adopted at larger, more well-established clinical schools. The evaluation component of the workshops, of course, only gathered immediate responses, and further systematic follow-up is planned. Finally, we did not attempt to compare our approach with more conventional, direct methods for reflection, which could be expected to yield useful insights. Our primary aim in attempting this novel and alternative approach was to encourage busy and assessment-focused medical students to take time out from their clinical schedule and, through fun, collaborative, and relatively efficient creative activities, explore the potential of reflective practice in all its guises. We believe our findings, however early and provisional, offer considerable promise when it comes to the value of such indirect and playful approaches to reflection through writing. To be able to generate such profound and relatable themes in a few sessions with very brief writing tasks was a significant outcome.

## Conclusion

The in-verse reflection approach appears to offer a feasible and stimulating opportunity to engage students with reflection about their learning while providing a sense of connectedness and an invaluable opportunity to share and discuss their clinical experiences and the process of professional identity formation. It does so through short, enjoyable, and structured creative exercises. The highly relevant and insightful nature of the creative outputs produced by the students point to the potential value of indirectness and playfulness when utilizing humanities approaches in medical education contexts. The described method adds to the repertoire of techniques to facilitate genuine reflection in medical students and can potentially assist medical schools in finding the necessary space in the curriculum for such activities.
